# Items

**Published:** 1897-12

**Authors:** 


### Items.

Beef Juice.—There is a palpable defect in beef extracts reduced to the solid, semi-solid and dry pulverulent condition. The necessary preparation of these by heat involves radical change in the organic matter, notably the albumin, an important constituent. The therapeutic utility becomes lessened and they are scarcely even auxiliary as food, whilst all stimulating property is reduced to the minimum of action. Natural beef juice—Wyeth's perfected beef juice—it is stated presents in an original organic state all the normal constituents of beef flesh, unaltered. The proprietors claim that this preparation is unquestionably the best of its class, and has an accredited superiority given to it by the most authentic and eminent medical sanction.

---

At the recent meeting of the American Public Health Association in Philadelphia, one of the most enjoyable and profitable of the many contributions to the special committee's arrangements for entertainment was a free tally-ho ride, given by the H. K. Mulford Company. The tally-ho's were equipped with every comfort and convenience and made frequent trips, passing through the most interesting sections of the city and a considerable portion of Fairmount Park and visiting Mulford's Biological Laboratory, where concentrated diphtheria antitoxin and allied products are prepared, and where every step in the preparation of antitoxin was observed by the guests.

These courtesies, it is needless to add, were highly enjoyed by every visitor and member of the Association who participated.

---

The Antikamnia Chemical Company is presenting the medical profession with a pocket medicine case that is both useful and unique. This company has succeeded in innovating a souvenir for its medical friends that will be of practical service to each recipient.

To add to its value there is enclosed in each case, in addition to the samples, a booklet entitled, A selection of practical prescriptions, being an alphabetically arranged list of prescriptions, for ready reference. The convenience with which the "Antikamnia Pocket Case" can be carried in the outside-top-vest-pocket will illustrate its further utility.

